# Verrucous Papillary Lesions: Dilemmas in Diagnosis and Terminology

**DOI:** 10.1155/2013/298249

**Published:** 2013-09-24

**Authors:** Thomas George Kallarakkal, Anand Ramanathan, Rosnah Binti Zain

**Affiliations:** ^1^Department of Oro-Maxillofacial Surgical & Medical Sciences, Faculty of Dentistry, University of Malaya, 50603 Kuala Lumpur, Malaysia; ^2^Oral Cancer Research & Coordinating Centre (OCRCC), Faculty of Dentistry, University of Malaya, 50603 Kuala Lumpur, Malaysia

## Abstract

Verrucous papillary lesions (VPLs) of oral cavity are diagnostically challenging as they include a spectrum of benign, potentially malignant, and frankly malignant lesions. A majority of the benign VPLs have viral aetiology and include commonly occurring squamous papilloma along with verruca vulgaris, focal epithelial hyperplasia, and condyloma. Current understanding of potentially malignant VPLs is perplexing and is primarily attributed to the use of confusing and unsatisfactory terminology. Clinically and histologically oral verrucous hyperplasia, a potentially malignant disorder, resembles oral verrucous carcinoma and may be indistinguishable from one another. The most reliable way to separate these entities on routine haematoxylin-eosin stained tissue sections is to recognize the exophytic growth patterns of oral verrucous hyperplasia from the combined exophytic and endophytic growth patterns associated with verrucous carcinoma. A review of the literature showed that there is a lot of confusion regarding the current clinical and histopathological guidelines to diagnose this potentially malignant entity. The criteria elaborated by different authors in establishing the diagnosis of oral verrucous hyperplasia are discussed in detail. A brief overview of the treatment modalities adopted is also discussed. The need for establishing a clear understanding of this potentially malignant entity is stressed as it may have far reaching implications on its management.

## 1. Introduction

Verrucous papillary lesions (VPLs) of the oral cavity are diagnostically challenging as they include a spectrum of benign, potentially malignant, and frankly malignant lesions. A majority of the benign VPLs have a viral aetiology and include the more commonly occurring squamous papilloma along with verruca vulgaris, focal epithelial hyperplasia, and condyloma [1]. Mucosal HPV types (HPV 6, 11, 13, 30, 32, 45, 52, 55, 59, 69, 72 and 73) have been isolated from these oral lesions [2]. Histopathologically, these benign lesions do not demonstrate any cellular atypia. It is sometimes difficult to distinguish these lesions, but clinical and certain histological features facilitate their diagnosis [3, 4]. 

Benign VPLs with known aetiologic factors will not be the focus of our discussion in this paper. Our current understanding of potentially malignant VPLs is perplexing and is primarily attributed to the use of confusing and unsatisfactory terminology. This may be best exemplified by verrucous hyperplasia, a potentially malignant disorder presenting as a verrucous or exophytic growth characterized by keratosis and/or varying grades of dysplasia [1]. Verrucous hyperplasia is a histopathological entity with clinical features that may be indistinguishable from a verrucous carcinoma [5]. The pathologist may fail to convey to the clinician the potentially malignant nature of verrucous hyperplasia due to the absence of overt features of dysplasia, and the clinician may subsequently consider this as a benign condition. This may become further established when reactive lesions such as inflammatory papillary hyperplasia may also be casually diagnosed as verrucous hyperplasia both clinically and histopathologically. 

## 2. Historical Background and Terminologies

The first ever documented evidence of a VPL dates back to 1941 when Fridell and Rosenthal reported a case of well-differentiated squamous cell carcinoma of the oral cavity as “papillary verrucoid carcinoma.” In 1948, Ackerman reported a series of thirty one similar cases and coined the term “verrucous carcinoma” [6]. Ackerman is credited with the recognition of distinctive clinical and microscopic features of verrucous carcinoma that he considered to be a variant of squamous cell carcinoma. A relatively high proportion of these lesions tend to involve the buccal mucosa in tobacco chewers [7]. These lesions are markedly exophytic and endophytic with a tendency to erode the underlying tissues including bone. Histomorphologic features include densely parakeratinized papillary surface, deep clefts in the epithelium, blunt and voluminous rete ridges with little or no dysplastic changes exhibiting a pushing border effect, and an intact basement membrane [8]. Ever since its original description within the oral cavity, there have been reports of similar lesions occurring at other sites including the larynx, perianal region, cervix, and glans penis [6]. Many of these later reports have remained true to the original description by Ackerman; however, challenges exist in recognizing an optimal therapeutic approach, the incidence of recurrence, and frequency of anaplastic transformation of verrucous carcinoma [9]. The general guidelines for the management of verrucous carcinoma of the head and neck recognize surgical excision as the primary treatment although the opinion is divided among investigators on the role of radiotherapy alone or as an adjunct to surgery as a treatment modality. The primary cause of concern is reflected in the views that irradiation of verrucous carcinoma is less effective and more likely to result in a recurrence with a more aggressive cancer through anaplastic differentiation [10]. Carcinoma cuniculatum is a variant of verrucous carcinoma and has also been described under an array of confusing terminologies including inverted verrucous carcinoma and oral florid papillomatosis to name a few [11].

Florid oral papillomatosis (FOP) is a rare disorder of the oral cavity and lips, characterised by the presence of multiple and multifocal papillomatous and verruciform growths that form confluent plaques and vegetation. It was originally recognized by Rock and Fisher [12] to describe multiple papillary lesions involving the mouth and larynx [13]. Malignant transformation has been reported in a subset of these cases. Strangely enough different, terminologies such as verrucous hyperplasia, verrucous leukoplakia, and papillomatosis mucosae carcinoides have been used synonymously to describe this lesion [14]. The histopathologic feature of FOP consisting of papillomatous and acanthotic as well as partially keratinized epithelium with elongated rete ridges is distinct from a verrucous carcinoma [14]. However, in the older literature, this lesion is considered to be synonymous with verrucous carcinoma [8]. 

**Figure 1:**
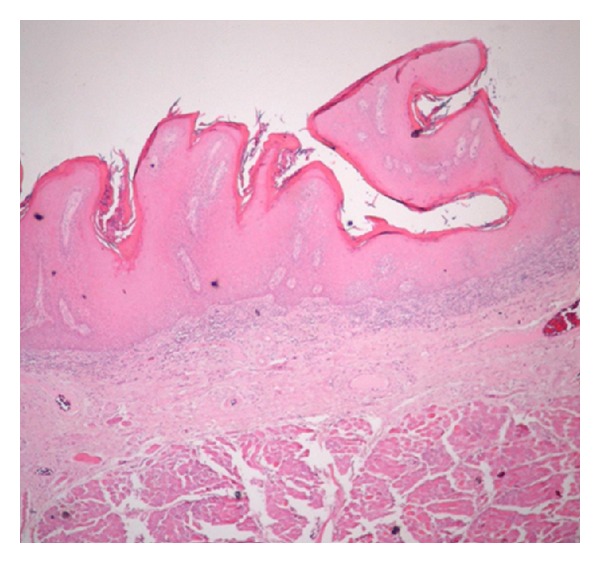
Photomicrograph of verrucous hyperplasia showing sharp surface projections (original magnification 4x, H and E stain).

**Figure 2:**
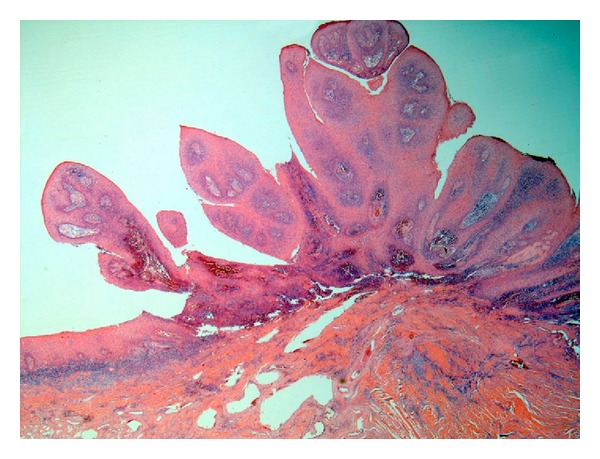
Photomicrograph of verrucous hyperplasia showing blunt surface projections (original magnification 4x, H and E stain).

**Figure 3:**
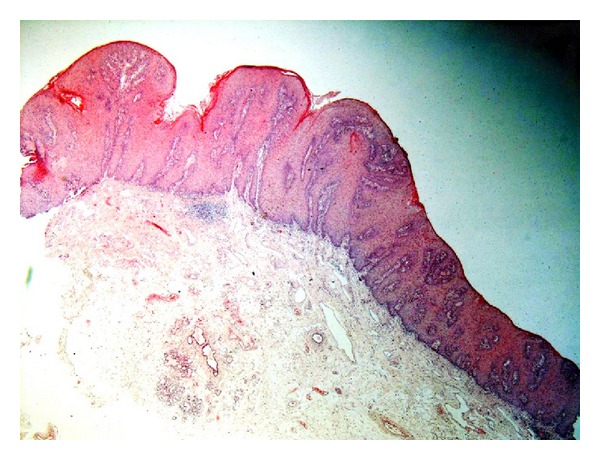
Photomicrograph of verrucous hyperplasia showing blunt surface projections (original magnification 4x, H and E stain).

Ackerman and McGavran [15] introduced the term “verrucous hyperplasia” to describe a condition that closely resembles verrucous carcinoma clinically and histologically. A subsequent review by Adkins and Monsour [16] concluded that an entity described as verrucous leukoplakia by many authors may actually correspond to some forms of verrucous hyperplasia [5]. Shear and Pindborg were the first to perform a detailed clinical and histological analysis of verrucous hyperplasias of the oral mucosa and unified previously used terms such as verrucous leukoplakia under this distinct histologic subset within the leukoplakia family of clinically identified lesions [17]. Clinically verrucous hyperplasias have been classified into two variants, a sharp variety (Figure 1) comprising long, narrow, and heavily keratinized verrucous processes which appears white as result of heavy keratinization. This entity may represent the form referred to as verrucous leukoplakia by many authors. The second clinical variant is a blunt variety (Figures 2 and 3) consisting of verrucous processes that are broader, flatter, and not heavily keratinized. In a majority of cases, areas of homogeneous leukoplakia are an integral component of the lesion and of the mucosa elsewhere in the mouths of the same patients [5]. Histologically, epithelial dysplasia is a prominent feature in verrucous hyperplasias and these lesions have been found to be juxtaposed with verrucous carcinoma and squamous cell carcinoma in a significant percentage of patients [17]. We propose that all lesions whether they are of the blunt/sharp-type should be relabelled as oral verrucous leukoplakia clinically. However, confusion persists with regard to the blunt-type lesions which are red, and it may not be possible to categorize them as verrucous leukoplakia. It is recommended that all these lesions should be diagnosed histologically as verrucous hyperplasia based on the following criteria subject to a consensus. The proposed histopathologic criteria for diagnosis of oral verrucous hyperplasia are as follows:

- long and narrow heavily keratinized verrucous processes or broad and flat verrucous processes that are less keratinized;
- absence of invasion of the hyperplastic epithelium into the lamina propria as compared with the adjacent normal mucosal epithelium;
- presence of cytologic/architectural features of dysplasia.

Oral verruciform leukoplakia is not firmly established in the literature. This terminology was proposed by Wang et al. to denote a subset of oral verrucous hyperplasias that strongly resemble verrucous leukoplakia clinically [18].

## 3. Diagnostic Dilemmas

### 3.1. Clinical Spectrum of Oral Verrucous Hyperplasia

It has been observed that leukoplakias may evolve through verrucous hyperplasias, verrucous carcinomas, and eventually squamous cell carcinomas [5]. A similar observation by Slootweg and Muller led them to hypothesize that verrucous hyperplasias and verrucous carcinomas represent a spectrum of the same process which represents a ubiquitous premalignant change in the whole oral mucous membrane [19]. The characteristic histologic feature that distinguishes a verrucous carcinoma is the presence of microscopic verrucous projections and endophytic epithelial extensions into the underlying lamina propria of which the latter are conspicuously absent in verrucous hyperplasia [17]. Hansen et al. [20] in his long term study on proliferative verrucous leukoplakia (PVL) considered verrucous hyperplasia and verrucous carcinoma as intermediate clinicopathological stages in its spectrum. This was subsequently confirmed by Batsakis et al. [21]. Originally described by Hansen et al. [20], PVL is a recognized specific type of nonhomogeneous leukoplakia with an extremely high propensity for malignant transformation [22]. Verrucous carcinomas exist within the histologic continuum ranging from benign squamous hyperplastic lesions and proliferative verrucous lesions to invasive squamous cell carcinoma. Distinguishing verrucous carcinoma from these similar benign and malignant processes may be difficult. The belief by earlier researchers that verrucous carcinomas may evolve into a conventional invasive squamous cell carcinoma may be due to presence of small foci of squamous cell carcinoma in those lesions with dominant features of verrucous carcinoma. Some investigators consider these verrucous squamous carcinomas to be “hybrid” forms of verrucous carcinoma or a squamous cell carcinoma with verrucoid features [10]. We firmly believe that addition of new terminologies may add to further confusion, and therefore, the two entities—verrucous carcinoma and squamous cell carcinoma—should remain independent. It is strongly recommended that surgical specimens of verrucous carcinoma should be thoroughly sampled to avoid missing an occult focus of conventional squamous cell carcinoma. This will provide valuable information on management as these two lesions have a different prognosis.

### 3.2. Clinical Variants of Oral Verrucous Hyperplasia

Wang et al. [18] discussed in detail the clinicopathologic features and behaviour of verrucous hyperplasia in sixty Taiwanese patients. Contrary to the earlier accepted histological subtypes as originally proposed by Shear and Pindborg, Wang reclassified these lesions into (1) plaque-type and (2) mass-type based on their histological features. The histologic criteria for diagnosis were primarily epithelial hyperplasia with parakeratosis or hyperkeratosis and a verrucous surface. An absence of invasion of the hyperplastic epithelium into the lamina propria as compared with the adjacent normal mucosal epithelium was an additional important histologic criterion for diagnosis. A surface keratin layer of >40 *μ* thickness was accepted as a differentiator between the two subtypes. Lesions exhibiting a verrucous surface with single or multiple protruding masses of epithelial growth showing minimal connective tissue cores and a surface keratin thickness of <40 *μ* were designated as the mass type. The plaque-type lesions demonstrated a verrucous surface, epithelial hyperplasia, and a surface keratin thickness of >40 *μ*. Clinically, the mass-type verrucous hyperplasia manifested as single or multiple mass-like verrucous whitish pink lesions while the plaque-type lesions appeared as whitish verrucous plaques. Dysplasia was not a determinant in the diagnosis of these lesions. However, in this study, the plaque-type lesions exhibited a greater frequency of epithelial dysplasia [18]. 

Wang et al. remarked that they had difficulty in correlating the clinical and histopathological features of the plaque-type lesions. The histopathological features of these lesions conferred with their criteria but, a subsequent clinical reevaluation led them to reclassify these lesions as oral verruciform leukoplakia. Thus, they concluded that the terminology oral verrucous hyperplasia should be used to denote the mass-type lesions both clinically and histologically. They also suggested that the plaque-type lesions should be clinically classified as oral verruciform leukoplakia and histologically as verruciform hyperplasia [18].

Our literature review from Taiwan also confirms that there is a general consensus among the various authors there regarding the clinical and histological characteristics of oral verrucous hyperplasia which is in agreement with guidelines proposed by Wang et al. as described earlier [23, 24]. There does not seem to be any differences of opinion regarding the potentially malignant nature of oral verrucous hyperplasias and its association with high risk habits such as tobacco and areca quid chewing and cigarette smoking. The primary sites of involvement include the buccal mucosa, vestibular mucosa, gingiva, and alveolar mucosa which are a reflection of a direct cause and effect relationship associated with risk habits [5, 18, 25, 26]. Clinically, they are manifested as white to whitish pink lesions that can be attributed to variations in the degree of keratinization. 

The clinical appearance of these lesions has not generally been well characterised as much emphasis has been laid on the verrucous/exophytic nature of these lesions with little attention being given to colour variation. Moreover, these lesions are considered to be clinically indistinguishable from verrucous carcinomas which are generally white or greyish white in colour [8, 27, 28]. A significant association with leukoplakia has been stressed in the literature. These areas of homogeneous leukoplakia occur adjacent to oral verrucous hyperplasia and histologically represent hyperkeratosis and epithelial dysplasia. This as well as evidence from long term follow up of patients with oral leukoplakia suggests that oral verrucous hyperplasia actually represents a process in continuum [5, 29]. Recognition of this feature is subdued in many of the reports from Taiwan where the lesions are primarily described as either the mass or the plaque types. A combination type lesion with a peripheral plaque and a central mass reported in the Taiwanese literature may be indicative of the associated leukoplakia [18, 24]. This needs to be addressed as reports from Taiwan may represent a subset of verrucous hyperplasia where changes in the adjacent mucosa are less pronounced. This has to be approached with caution bearing in mind the concept of field cancerization where abnormal, hyperplastic, and often atypical epithelium clinically visible as leukoplakia or atrophic epithelium may represent an area that has been preconditioned by a carcinogen to develop into a malignancy [30].

## 4. Histopathological Features of Oral Verrucous Hyperplasia

Histologically, oral verrucous hyperplasia resembles oral verrucous carcinoma and may be indistinguishable from one another. The most reliable way to separate these entities on routine haematoxylin-eosin stained tissue sections is to recognize the exophytic growth pattern of oral verrucous hyperplasia from the combined exophytic and endophytic growth pattern associated with a verrucous carcinoma. In oral verrucous carcinoma, the projections of neoplastic epithelium are seen deep to the adjacent uninvolved epithelium, whereas, in oral verrucous hyperplasia, they are seen only at the same level as the adjacent epithelium. However, separation of these lesions is often obscured by small biopsies, poorly orientated specimens, and biopsies that fail to demonstrate the lesion margins [27]. The aforementioned histological features of oral verrucous hyperplasia are unanimously recognized as a prerequisite for its diagnosis, whereas dysplasia is not. Epithelial dysplasia with a propensity for moderate dysplasia has been reported in a majority of cases of verrucous hyperplasia ranging from 18 to 68% [5, 18, 26]. In a hospital based follow-up study from Taiwan where oral verrucous hyperplasia is very common, the malignant transformation rate was estimated at 20% in a cohort of forty-four male subjects with verrucous hyperplasia. This was only second to epithelial dysplasia which exhibited the highest rate of malignant transformation of 24% [25]. These estimates may further add to the confusion as it has not been clearly stated whether cases classified as epithelial dysplasia also included cases of verrucous hyperplasia with dysplasia in the first place. 

## 5. Treatment of Oral Verrucous Hyperplasia

Poswillo is of the opinion that oral verrucous hyperplasia and verrucous carcinoma should be managed similarly because of the significant overlap in their clinicopathologic features [31]. Many reports consider oral verrucous hyperplasia as a potentially malignant disorder [18, 25]. However, it has not been listed so by the WHO [32]. It is well established that verrucous carcinoma is a low grade malignancy. It is also clear that verrucous hyperplasia is a forerunner of verrucous carcinoma, and transition to the latter is quite consistent. Hence there is an opinion that the two lesions should be managed identically [33]. Verrucous carcinoma has been treated with different modalities such as excision with or without radical surgery, chemotherapy, radiation, or a combination of these modalities [29]. Surgery is the most common treatment modality, while the use of radiotherapy is controversial. The conventional treatment of oral verrucous hyperplasia has been total surgical excision. Recurrence and/or transformation of oral verrucous hyperplasia to either verrucous carcinoma or conventional SCC have been reported after surgical intervention. Shear and Pindborg reported recurrence in four of their patients with lesions showing both verrucous hyperplasia and verrucous carcinoma [5]. This may be subject to the use of strict criteria for defining recurrence and differentiating it from residual lesions. Wide surgical excision of the primary verrucous lesion with adequate mucosal and soft-tissue margin is necessary to avoid local recurrence [33].

## 6. Conclusion

It is evident from our discussion that clearer guidelines for recognizing the clinical and histopathological features should be established to diagnose oral verrucous hyperplasia. It is proposed that both the sharp and the blunt varieties of oral verrucous hyperplasia as originally recognized by Shear and Pindborg and subsequently relabelled as the plaque-type and mass-type lesions should be best diagnosed clinically as a non-homogeneous leukoplakia or more specifically as verrucous leukoplakia [32]. Following histological evaluation, the lesions may be further characterised as oral verrucous hyperplasia subject to a consensus.
